# Tension band high-strength suture combined with absorbable cannulated screws for treating transverse patellar fractures: finite element analysis and clinical study

**DOI:** 10.3389/fbioe.2024.1340482

**Published:** 2024-03-07

**Authors:** Feifan Xiang, Yukun Xiao, Dige Li, Wenzhe Ma, Yue Chen, Yunkang Yang

**Affiliations:** ^1^ The State Key Laboratory of Quality Research in Chinese Medicine, Macau University of Science and Technology, Macau, China; ^2^ Department of Orthopedic, Affiliated Hospital of Southwest Medical University, Luzhou, China; ^3^ Department of Nuclear Medicine, Affiliated Hospital of Southwest Medical University, Luzhou, China; ^4^ Nuclear Medicine and Molecular Imaging Key Laboratory of Sichuan Province, Luzhou, China; ^5^ Institute of Nuclear Medicine, Southwest Medical University, Luzhou, China

**Keywords:** transverse patellar fractures, absorbable cannulated screws, ultrabraid highstrength suture, tension band, finite element analysis, retrospective clinical study

## Abstract

**Objective:** Few reports exist on the treatment of transverse patellar fractures (TPFs) using absorbable cannulated screws and high-strength sutures, and most screws and sutures lack good biomechanics and clinical trials. Therefore, this study aimed to demonstrate the biomechanical stability and clinical efficacy of tension-band high-strength sutures combined with absorbable cannulated screws (TBSAS) in treating TPFs (AO/OTA 34 C1).

**Methods:** Finite element models of five internal fixation schemes were established: tension-band wire with K-wire (TBW), TBW with cerclage wire (TBWC), TBW with headless pressure screws (TBWHS), TBW with full-thread screws (TBWFS), and TBSAS. We comprehensively compared the biomechanical characteristics of the TBSAS treatment scheme during knee flexion and extension. Forty-one patients with TPFs in our hospital between January 2020 and August 2022 were retrospectively enrolled and divided into the TBSAS (*n* = 22) and TBWC (*n* = 19) groups. Clinical and follow-up outcomes, including operative time, visual analog scale (VAS) pain score, postoperative complications, Bostman score, and final knee range of motion, were compared between both groups.

**Results:** Finite element analysis (FEA) showed that TBWHS and TBWFS achieved the minimum mean fracture interface relative displacement during knee flexion (45°, 0–500 N bending load) and full extension (0°, 0–500 N axial load). There was no significant difference between TBSAS (0.136 mm) and TBWC (0.146 mm) during knee flexion (500 N); however, TBSAS displacement was smaller (0.075 mm) during full extension (500 N). Furthermore, the stress results for the internal fixation and the patella were generally lower when using TBSAS. Retrospective clinical studies showed that the TBSAS group had a shorter operative time, lower VAS pain score at 1 and 2 months postoperatively, better Bostman knee function score at 3 and 9 months postoperatively, and better final knee joint motion than the TBWC group (all *p* < 0.05). There were five cases (26.3%) of internal fixation stimulation complications in the TBWC group.

**Conclusion:** TBSAS demonstrated excellent safety and effectiveness in treating TPFs. It is sufficient to meet the needs of TPF fixation and early functional exercise and effectively reduces metal internal fixation-induced complications and secondary surgery-induced trauma.

## 1 Introduction

Patellar fracture is a common intra-articular fracture in clinical practice, with transverse patellar fracture (TPF) being the most common, accounting for approximately 23% of cases (Larsen et al., 2016; Henrichsen et al., 2018). The patella is the largest sesamoid bone in the human body and plays a vital role in transmitting the strength of the quadriceps muscle and in composing a knee extension device (Martin et al., 2019; Jirangkul and Kosiyatrakul, 2021). Patellar fractures cause serious damage to knee extension. Therefore, the treatment goals for patellar fractures are anatomical reduction of the fracture and articular surface and stable fixation, allowing early functional exercise of the knee joint (Ma et al., 2022). Surgical treatment is necessary when the fracture is displaced by > 3 mm or when the joint is inconsistent by > 2 mm (Steinmetz et al., 2020).

There are many surgical options for TPFs, with tension-band wire with K-wire (TBW) being the most widely used (Kruse et al., 2022). The tension band technique converts the patellar surface tension generated by the extensor muscle during knee flexion into the axial compression force of the patellar fracture surface, thereby promoting bone healing and demonstrating good efficacy (Jia et al., 2022). However, owing to the specific anatomic location of the patella, it is associated with a higher overall complication rate (approximately 52%) (Li et al., 2022), including Kirschner needle displacement, tension band breakage, symptomatic hardware, and infection (Lee et al., 2021; Trinchese et al., 2021). The modified regimen of titanium-cannulated screws may have better biomechanical characteristics and stability and can provide a direct compression force between the fracture fragments. Currently, there are mainly headless pressure screws and full-thread screws (Chen et al., 2019). However, clinical complications such as fixation failure (7.5%), postoperative infection (1.5%), and symptomatic implants (23%) remain (Hoshino et al., 2013; Jia et al., 2022). Therefore, another surgery or revision is required to remove the internal fixation, which increases the patient’s pain.

In recent years, non-metallic implants have received significant attention and have advanced rapidly, and there are new treatment options for TPFs. Poly lactic-co-glycolic acid (PLGA) is one of the most widely used biodegradable forged composite materials, with good biological activity, biocompatibility, and high mechanical strength (Kobielarz et al., 2020; Rocha et al., 2022). The PLGA absorbable cannulated screw can be directly combined with bone, completely replaced with natural bone, and finally hydrolyzed into alpha-hydroxyl acid, which is fully absorbed in approximately 2 years. Furthermore, steel wire can be replaced with Ultrabraid™ #2 suture (Smith and Nephew, Andover, MA, United States), a nonabsorbable high-strength suture that has strong biomechanical benefits and is widely used in treating tendon and ligament rupture, meniscus injury, and fractures (Liu et al., 2020; Taha et al., 2020). This may provide an effective internal fixation scheme for TPFs. However, there are few reports on the treatment of TPFs using absorbable cannulated screws and high-strength sutures, and most screws and sutures lack good biomechanics and clinical trials (Wright et al., 2009; Sayum Filho et al., 2021). Finite element analysis (FEA) can provide quantitative biomechanical information on orthopedic implants and improve the understanding of the mechanical behavior of implants and bone-implant interactions (Zeng et al., 2020).

Therefore, to further improve the surgical efficacy of TPFs and reduce complications, the present study combined FEA with a retrospective clinical study to evaluate the safety and efficacy of tension-band high-strength sutures combined with absorbable cannulated screws (TBSAS) in treating TPFs.

## 2 Material and methods

### 2.1 FEA: establishment of the TPF model

**FIGURE 1:**
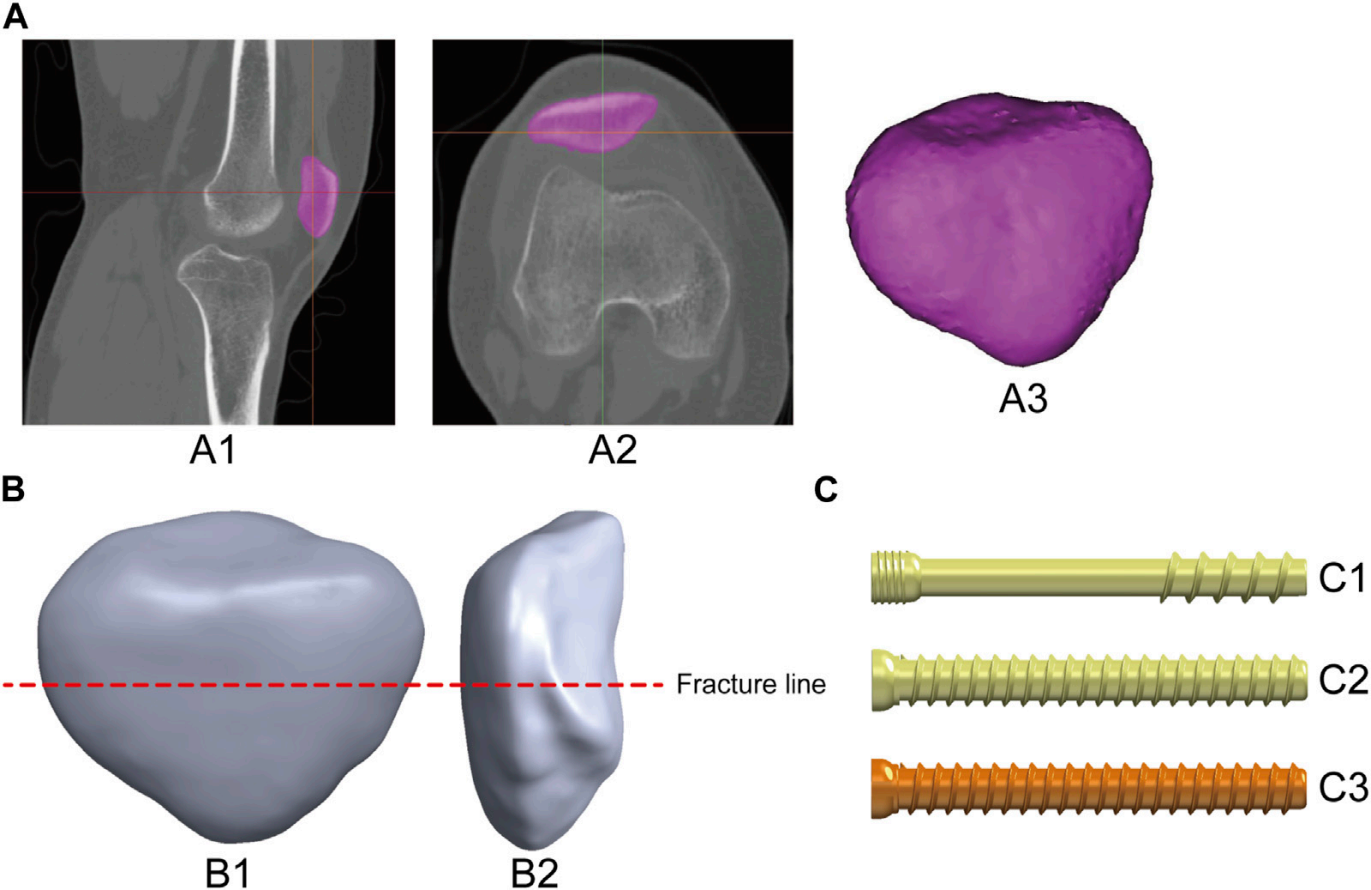
Production of the patella and internal fixation model: **(A)** 3D patella model extracted from image data, A1-A2: CT image of the knee joint; A3: 3D patella model. **(B)** Establishing the TPF model, B1-B2: adem position. **(C)** Construct three kinds of cannulated screw internal fixation models, C1: Headless pressure screw (Waston Medical Instrument Co., Ltd., China); C2: Full thread screw (Waston Medical Instrument Co., Ltd., China); C3: Absorbable screw (Bioretec Ltd., Finland).

This study was approved by the medical ethics committee of our hospital, and all participants provided informed consent. The patellar image data of a healthy volunteer (age: 41 years, height: 176 cm, weight: 73 kg) were collected using spiral computed tomography (CT) (GE Medical Systems 64-slice spiral CT scanner, layer thickness: 0.5 mm) and saved in Digital Imaging and Communications in Medicine format. The cortical and cancellous bone structures of the patella were extracted using threshold segmentation, region growing, and other commands in Mimics Research 21 (Materialize, Belgium) to build a three-dimensional model of the patella (Figure 1A) (Yuan et al., 2022). Remeshing, wrapping, and smoothing were performed using Geomagic Wrap 2021 (Geomagic, NC, United States) (Mao et al., 2023). Finally, the TPF model (AO/OTA 34C1) was established using SolidWorks 2021 (Dassault, France) (Chang et al., 2023) (Figure 1B).

### 2.2 Establishment of the internal fixation model

**FIGURE 2:**
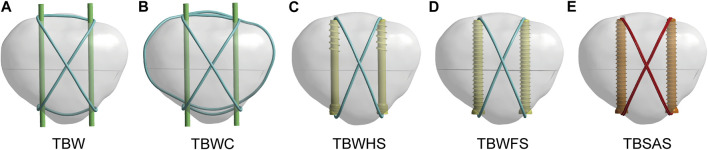
TPF internal fixation assembly model: **(A)** Tension band wire with K-wire (TBW). **(B)** TBW with cerclage wire (TBWC). **(C)** TBW with headless pressure screws (TBWHS). **(D)** TBW with full-thread screws (TBWFS). **(E)** Tension band high-strength sutures combined with absorbable cannulated screws (TBSAS). (Blue: steel wire; Green: Kirschner wire; Yellow: titanium screw; Red: sutures; Orange: absorbable screw).

In this study, five types of internal fixation models were selected to fix TPFs to fully compare and discuss the biomechanical effects of TBSAS. Three types of cannulated screws were constructed using the SolidWorks 2021 software (Figure 1C). Fixation was then performed according to the patellar fracture model and the standard surgical protocol (Figure 2). The patients were divided into control groups, including TBW, TBW with cerclage wire (TBWC), TBW with headless pressure screws (TBWHS), and TBW with full-thread screws (TBWFS), and an experimental group, TBSAS. The Kirchner needle and wire diameters were 2 and 1 mm, respectively. Screws with a diameter of 4.5 mm and a length of 40 mm were selected, and high-strength sutures with a diameter of 0.58 mm and made of double strands were selected. From the distal to the proximal end of the fractured patella, two Kirschner needles or screws were placed parallel in the middle third of the patella using “Boolean operation,” 5 mm from the articular surface (Ling et al., 2019).

### 2.3 Finite element structural analysis

**FIGURE 3:**
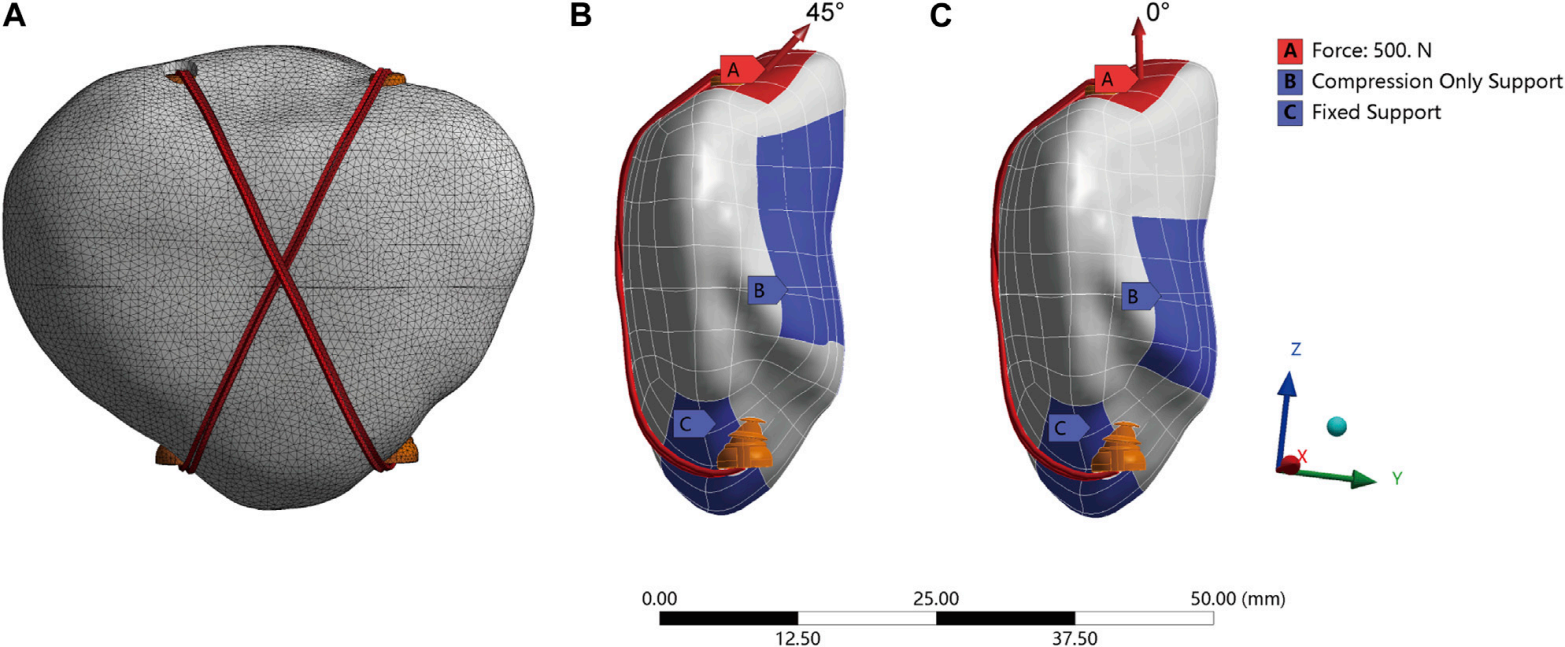
The setting of patella model finite element analysis: **(A)** Model mesh rendering. **(B,C)** Boundary and loading conditions of simulated knee flexion (45°bending load) and knee extension (0°axial load).

The physical models were simulated using ANSYS Workbench 2020 R1 (Swanson Analysis, Houston, PA, United States). The models were meshed using quadratic tetrahedral elements (Figure 3A). A convergence analysis was performed to ensure the stability and accuracy of the mesh state (Huang et al., 2023). Different field variables, such as the maximum von Mises stress and displacement, were <5% with no maximum stress point (Supplementary Information S1). The average sizes of the unit mesh of the patella, Kirschner needle, steel wire, screws, and suture were 0.7, 0.5, 0.5, 0.5, and 0.4 mm, respectively. All materials were modeled as homogeneous and linearly isotropic. The material properties used in this study are as previously described (Bartolin et al., 2021; Du et al., 2022; Xue et al., 2022). The parameters of the various materials are listed in Table 1.

**TABLE 1:** Model material parameters.

Material	Young’s modulus (MPa)	Poisson’s ratio
Cortical bone	10,000	0.3
Cancellous bone	840	0.29
Kirschner wire	200,000	0.3
Screw wire	100,000	0.29
Titanium alloy	110,000	0.3
Absorbable screw	5,500	0.3
Ultrabraid suture	3,000	0.4

### 2.4 Boundary and loading conditions

To simulate the actual situation of the contact relationship, all contact types were set within the Coulomb friction law: bone–bone (friction coefficient: μ = 0.45), bone–implant (μ = 0.3), and implant–implant (μ = 0.2) (Chen et al., 2019; Zeng et al., 2020; Xue et al., 2022). Cortical and cancellous bones were used as binding contacts. No prestrain was applied to the screws between the two bone fragments. To simulate the force of the quadriceps muscle during the extension and flexion of the knee joint (Chen et al., 2022), 0° axial load and 45° bending load (0–500 N) were applied on the tip of the patella, and the contact of the patellofemoral joint surface was simulated by setting the “Compression Only Support” boundary condition (Chen et al., 2019). During the analysis, the nodes on the distal surface of the patella were constrained to 0° of freedom, simulating the steady pull of the patellar ligament to prevent rigid-body movement (Figures 3B, C).

### 2.5 Retrospective clinical study

The clinical and follow-up data of 41 patients with TPF admitted to our hospital between January 2020 and August 2022 were retrospectively analyzed. All participants provided informed consent. The TBSAS and TBWC groups included 22 and 19 patients, respectively (Table 2). The inclusion criteria were as follows: 1) CT or X-ray diagnosis of TPF, 2) acceptance of TBWC or TBSAS, 3) age >18 years, and 4) informed consent and complete clinical data. The exclusion criteria were as follows: 1) other types of patellar fracture; 2) patellar fracture caused by infection, tumor, or metabolic disease; 3) severe structural damage around the patella; and 5) no or <12-month follow-up.

**TABLE 2:** Baseline characteristics of the enrolled patients.

Variables	TBWC (*n* = 19)	TBSAS (*n* = 22)	*p*-value
Age (years) (mean ± SD)	54.63 ± 8.02	52.82 ± 8.92	0.500
Gender			0.752
Male	12 (63%)	12 (55%)	
Female	7 (37%)	10 (45%)	
BMI group, no. (%)			0.308
Normal	8 (42%)	10 (45%)	
Overweight	10 (53%)	12 (45%)	
Obesity	1 (5%)	0	
Fracture side			0.938
Left	8 (42%)	9 (41%)	
Right	11 (58%)	13 (59%)	
Injury mechanism, no. (%)			0.763
Tumble	10 (52%)	14 (64%)	
High fall injury	6 (32%)	5 (23%)	
Car accident	3 (16%)	3 (13%)	
Follow-up (months)	14.47 ± 2.29	14.55 ± 2.54	0.925

### 2.6 Surgical procedure

**FIGURE 4:**
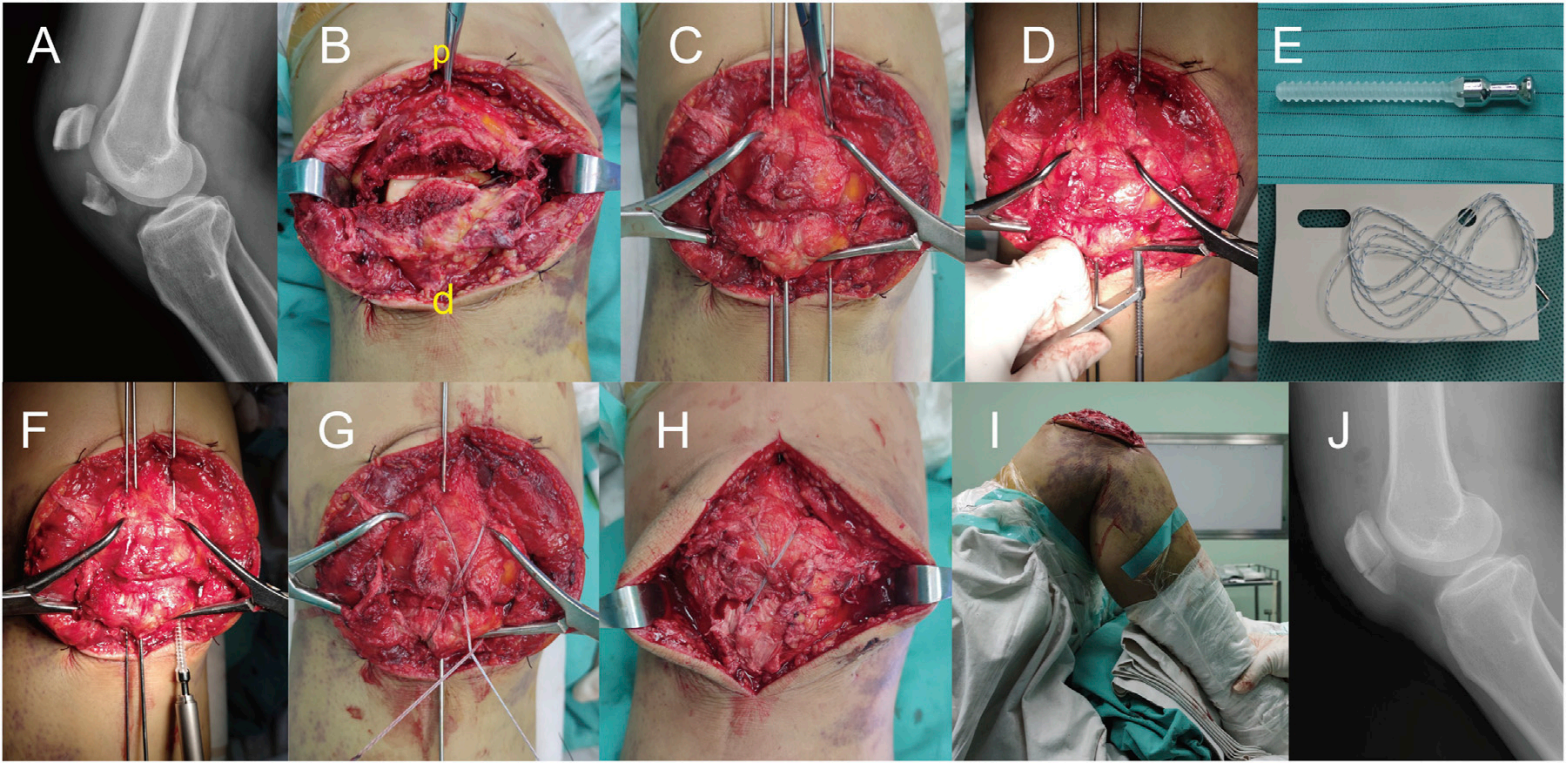
Intraoperative treatment of a transverse patellar fracture with TBSAS. **(A)** Preoperative X-ray image; **(B)** Exposed fracture site (p: proximal end of the patella; d: distal end of the patella); **(C)** Reduction fracture; **(D)** Expand the tunnels; **(E)** Absorbable cannulated screw and Ultrabraid high-strength suture; **(F)** Screw in two 4.5 mm absorbable cannulated screws; **(G)** Bound and fix the double-strand high-strength suture; **(H)** Condition after fixation; **(I)** Passive flexion and extension of the knee joint; **(J)** Postoperative X-ray image.

The two groups received treatment for TPFs according to the standard clinical surgical protocol to ensure patellar stability during each operation. All procedures were performed by the same veteran orthopedic trauma surgeon. In the TBSAS group, Kirschner wires and reduction forceps were temporarily fixed after careful reduction of the fracture to ensure a smooth patellar articular surface. Two 4.5 mm absorbable cannulated screws were screwed from the distal to the proximal end. Receding the Kirchner needles, the double-strand Ultrabraid high-strength suture was passed through the absorbable cannulated screws and was bound and fixed using a tension band and “NICE” junction technologies. Furthermore, patellar fixation can be further strengthened using Ultrabraid suture or Ethibond #5 suture cerclage fixation. Passive flexion and extension of the knee joint were performed immediately during the operation to check the stability of fracture fixation and knee joint movement. The key intraoperative steps are shown in Figure 4.

Notably, strict postoperative management was ensured, including strengthening dressing changes and focusing on wound recovery. Postoperative radiographic examination was performed. Plaster fixation was not required postoperatively, and an adjustable brace could be worn to assist with functional exercises. One day postoperatively, the knee could begin to flex and extend, and semi-weight training could be started gradually. Full weight training could begin 1 month postoperatively, and strenuous exercise should be avoided for 3 months (the target motion angle is 90° at 1 month postoperatively).

### 2.7 Follow-up data collection

The operative times were recorded. Postoperative follow-up was conducted every 4 weeks after discharge. Radiographic review of the affected limb was completed, and knee activity, visual analog scale (VAS) pain score, Bostman knee function score, and patient satisfaction were recorded. Fracture healing was evaluated based on radiographic examination and clinical results, and the patient was instructed to perform functional knee exercises.

### 2.8 Statistical analysis

Data were analyzed using SPSS software (version 22.0; SPSS Inc., Chicago, IL, United States). Data are presented as mean ± standard deviation. After applying the Kolmogorov–Smirnov normality test, differences between groups were assessed using a one-way analysis of variance or an independent sample *t*-test. Statistical significance was set at ∗*p* < 0.05, ∗∗*p* < 0.01, and ∗∗∗*p* < 0.001.

## 3 Results

### 3.1 FEA: displacement of fractures

**FIGURE 5:**
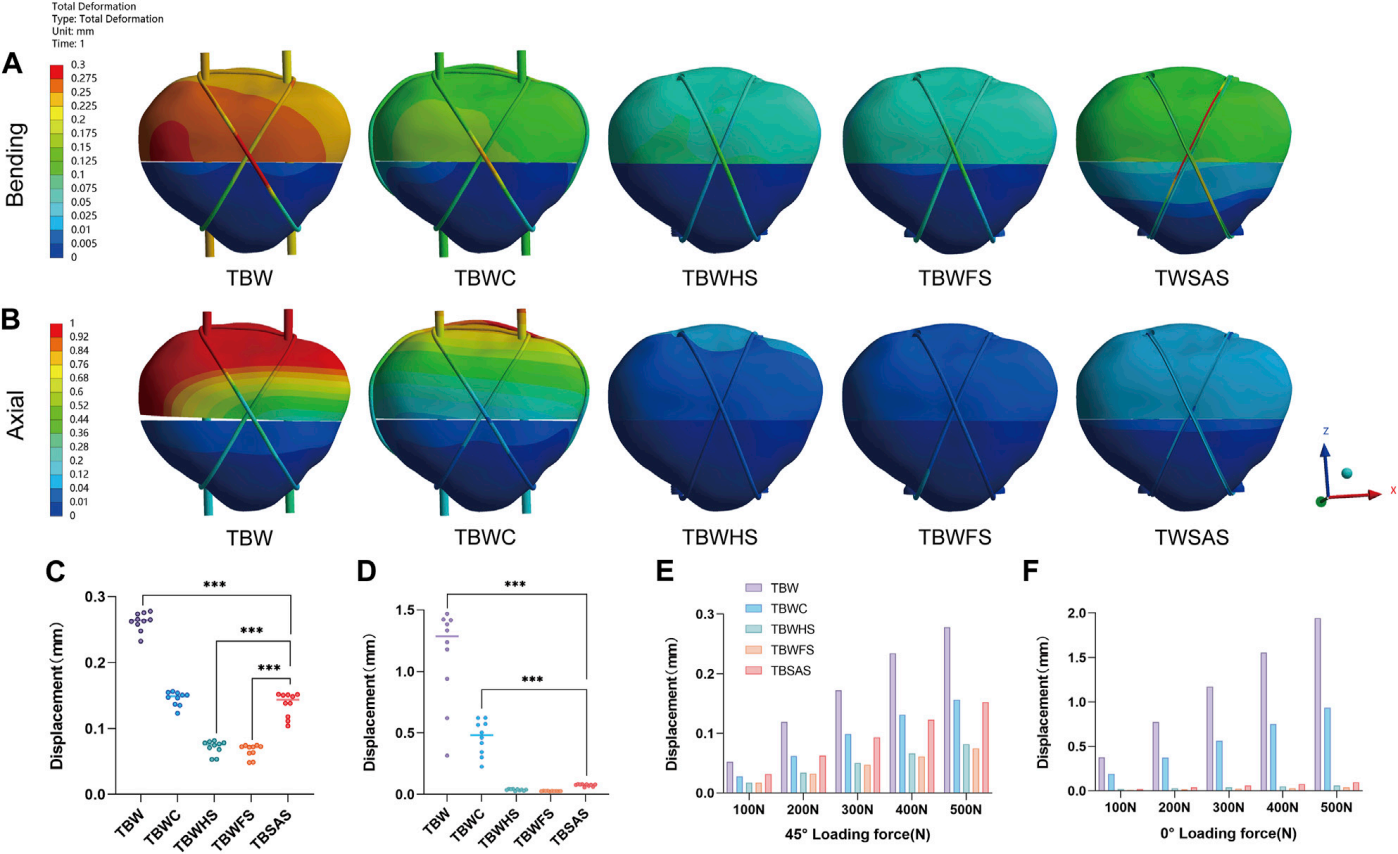
Displacements of the five models under bending and axial loads: **(A,B)** Displacement nephograms of the five models under 45° bending load and 0° axial load of 500N; **(C,D)** Statistical graphs of average fracture interface relative displacement of the five models under 45° bending load and 0° axial load of 500N; **(E,F)** Maximum bone displacements of the five models under 45° bending load and 0° axial load (0–500N).

Under a 45° bending load (0–500 N), the patellar fracture was angled backward. Ten points were uniformly selected at the proximal end of the patellar fracture interface to calculate the average relative displacement of the fracture end (Figure 5). The displacement of the fracture mass in all five models was relatively small. At 500 N, the mean fracture displacements of the two titanium screw groups were smaller than those of the TBSAS group (TBWHS: 0.072; TBWFS: 0.066 mm; TBSAS: 0.136 mm). Notably, no statistically significant difference was observed between TBWC (0.146 mm) and TBSAS. The displacement of the TBW (0.262 mm) was relatively large. However, under a 0° axial load (0–500 N), the patellar articular surface angled forward slightly. The mean fracture interface relative displacement of the patellar articular surface was calculated. In contrast, the interface relative fracture displacement was smaller in all three screw groups at 500 N (TBWHS: 0.037 mm; TBWFS: 0.027 mm; TBSAS: 0.075 mm) than in the TBW (1.132 mm) and TBWC (0.464 mm) groups.

### 3.2 Stress distribution on internal fixation

**FIGURE 6:**
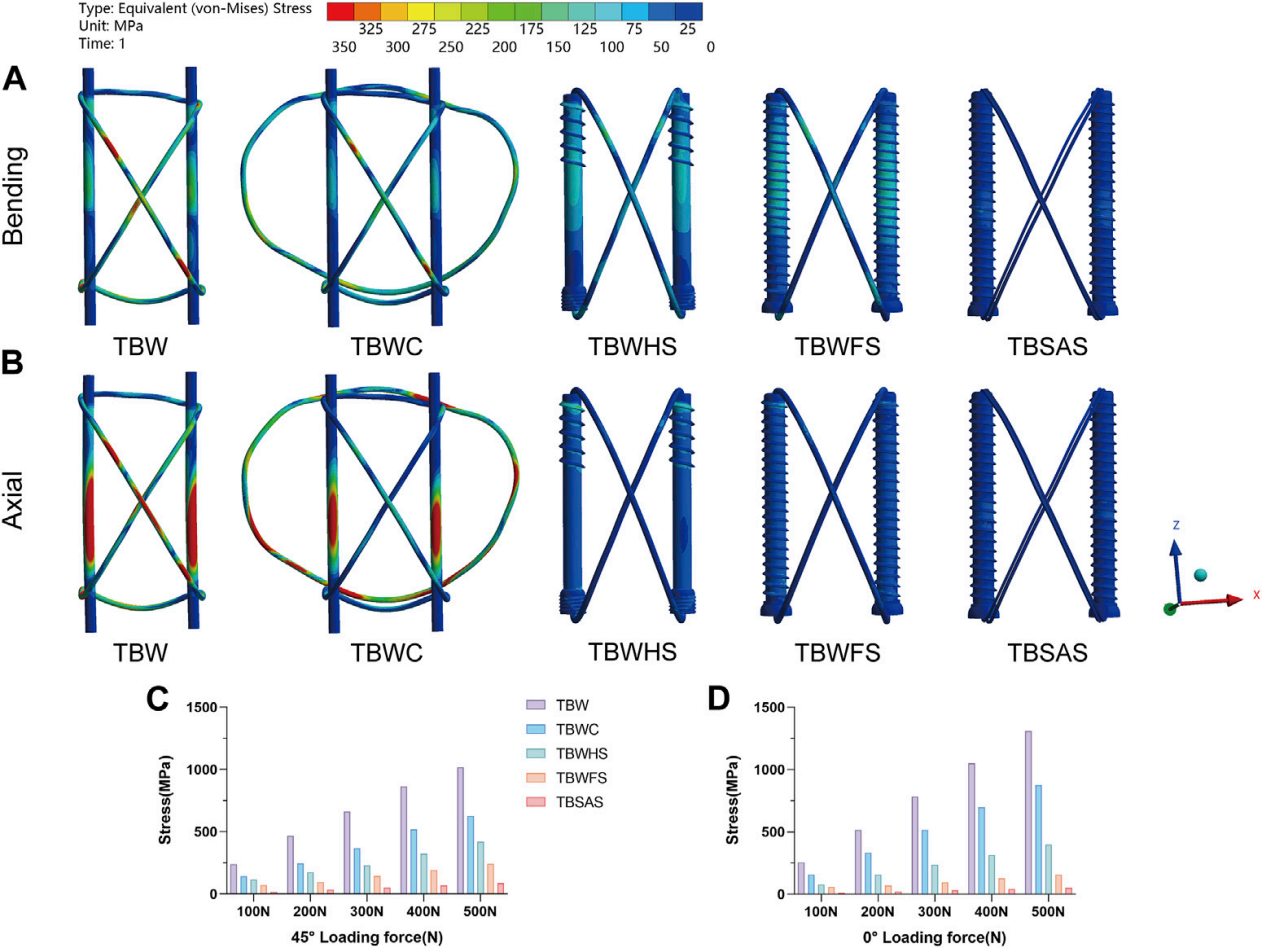
Stress distribution on internal fixation of the five models under bending and axial loads: **(A,B)** Stress distribution on internal fixation of the five models under 45° bending load and 0° axial load of 500N; **(C,D)** The maximum stresses on internal fixation of five models under 45° bending load and 0° axial load (0–500N).

Under a 45° bending load (0–500 N), stress concentration occurs mainly at the fracture site (Figure 6). At 500 N, the maximum von Mises stress of the five models decreased gradually in the following order: TBW (1,018.1 MPa), TBWC (628.19 MPa), TBWHS (418.8 MPa), TBWFS (242.21 MPa), and TBSAS (88.293 MPa). However, under a 0° axial load (0–500N), the stress concentrations in the three screw groups mainly occurred at the proximal thread of the screws. At 500 N, the maximum stress of the five models gradually decreased in the following order: TBW (1,310.8 MPa), TBWC (876.32 MPa), TBWHS (397.69 MPa), TBWFS (158.18 MPa), and TBSAS (53.201 MPa).

### 3.3 Stress distribution on the patella

**FIGURE 7:**
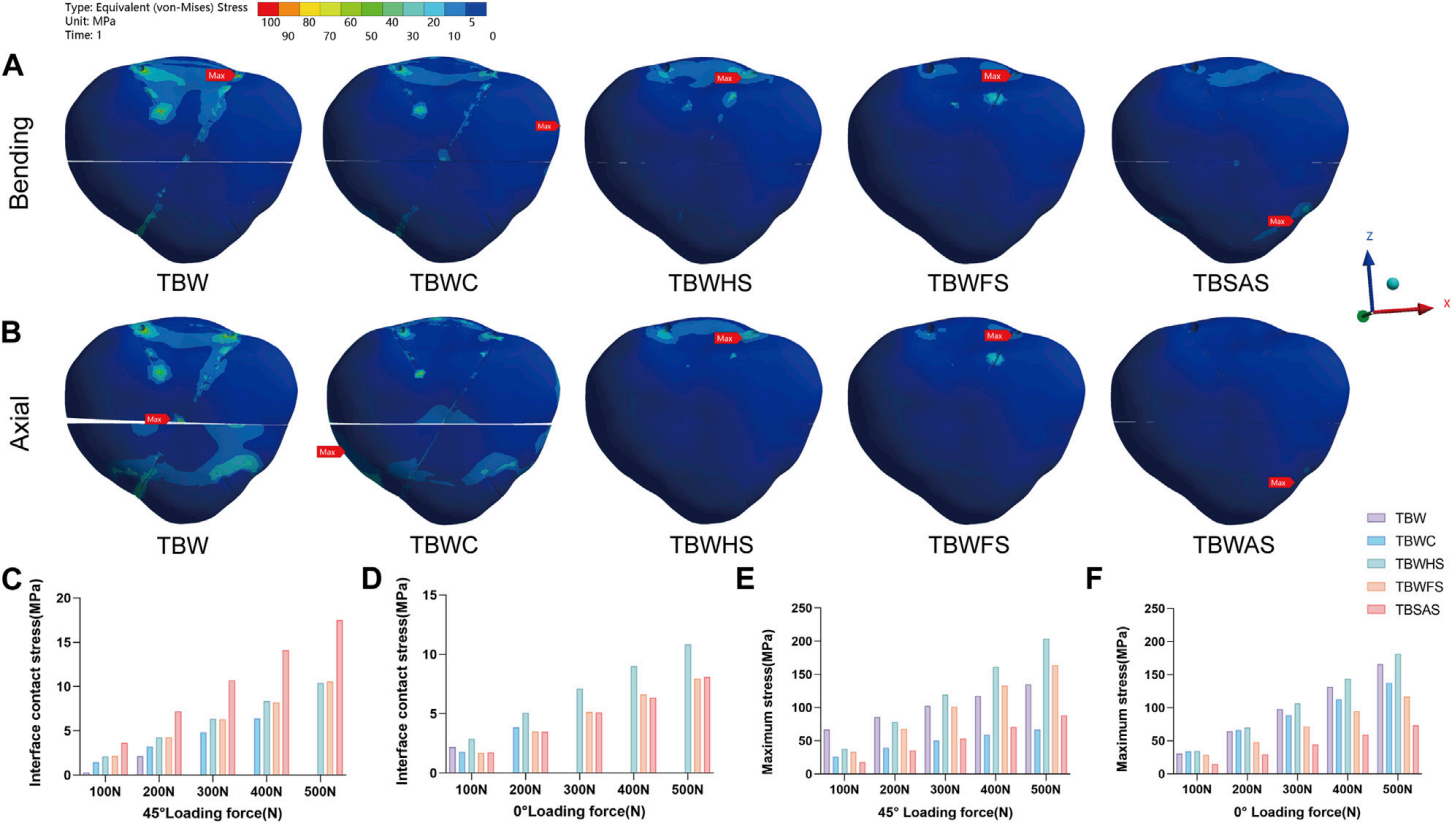
Stress distribution on patella of the five models under bending and axial loads: **(A,B)** Stress distribution on patella of the five models under 45° bending load and 0° axial load of 500N; **(C,D)** The maximum fracture interface contact stress of five models under 45° bending load and 0° axial load (0–500N) (y = 0 indicates separation of the fracture interface); **(E,F)** The maximum stresses on patella of five models under 45° bending load and 0° axial load (0–500N).

The stress distribution on the patella was mainly concentrated on the part in contact with the internal fixation (Figure 7). The magnitude trend of the patellar stress differed from that of the internal fixation stress, possibly owing to the friction contact of the threads in the three screw groups. Under a 45° bending load (500 N), the maximum von Mises stress from largest to smallest was as follows (Figures 7A, E): TBWHS (203.64 MPa), TBWFS (163.64 MPa), TBW (134.83 MPa), TBSAS (88.224 MPa), and TBWC (66.945 MPa). Under the 0° axial load (500 N), the maximum stress, from largest to smallest, was as follows (Figures 7B, F): TBWHS (181.62 MPa), TBW (165.88 MPa), TBWC (137.39 MPa), TBWFS (117.21 MPa), and TBSAS (73.591 MPa). As for the fracture interface contact stress (Figures 7C, D), all three groups of screws produced good interface compression. However, there was a gradual separation between TBW and TBWC, which was more significant at 0° axial loading.

### 3.4 Clinical outcomes

**FIGURE 8:**
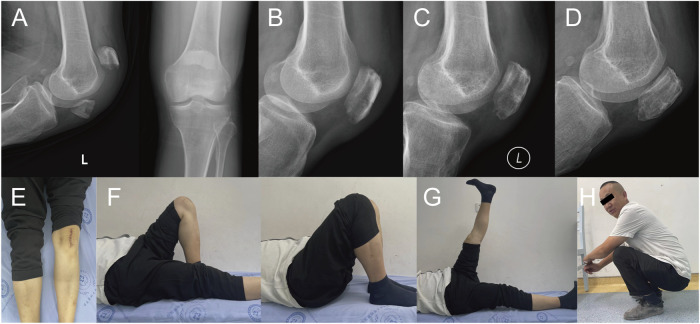
A 51-year-old male with a left TPF from a fall. X-rays: **(A)** Before the fracture. **(B)** The first day after surgery. **(C)** Three months after surgery. **(D)** Twelve months after surgery. **(E)** External observation; **(F–H)** Functional reexamination photos 1 year after surgery.

All included patients received standard surgical treatment within 48 h of admission and were discharged within 3–5 days postoperatively. Strict follow-up attention and guided functional exercises were ensured. In both groups, the average follow-up period was 14.51 months (range, 12–18 months), the fracture healing rate was 100%, and limb function recovery was satisfactory in most patients. In the TBWC group, the average operative time was 71.21 min, and the average VAS scores were 5.16 and 3.26 at 1 and 2 months postoperatively, respectively. The average Bostman scores were 21.53 and 25.53 at 3 and 9 months postoperatively, respectively, and the average final knee range of motion was 127.5°. In the TBSAS group, the average operative time was 53.95 min, and the average VAS scores were 3.14 and 1.32 at 1 and 2 months postoperatively, respectively. The average Bostman scores were 25.18 and 28.55 at 3 and 9 months postoperatively, respectively, and the average final knee range of motion was 131.5° (Table 3). Regarding postoperative complications, three patients in the TBWC group had postoperative redness and swelling at the incision site, which reduced after symptomatic treatment. Five patients treated with TBWC had persistent subcutaneous soft tissue irritation, which was relieved after removing the metal fixation 12 months postoperatively. A typical example is shown in Figure 8.

**TABLE 3:** Comparison of clinical data between TBWC and TBSAS in treating TPFs (mean ± SD).

Variables	TBWC (*n* = 19)	TBSAS (*n* = 22)	*p*-value
Duration of Surgery (min)	71.21 ± 9.78	53.95 ± 4.82	<0.05
VAS Score 1	5.16 ± 0.96	3.14 ± 0.83	<0.05
VAS Score 2	3.26 ± 1.05	1.32 ± 0.78	<0.05
Bostman Score 3	21.53 ± 2.04	25.18 ± 1.14	<0.05
Bostman Score 9	25.53 ± 1.68	28.55 ± 0.96	<0.05
Final ROM	127.5 ± 4.26	131.5 ± 1.37	<0.05

Note: VAS, Score 1 and VAS Score 2 represent VAS, scores at the one and 2 months postoperatively, respectively; Bostman Score 3 and Bostman Score 9 represent Bostman scores at the third and ninth months postoperatively, respectively. ROM: knee range of motion.

## 4 Discussion

At 500 N, during knee flexion (45° bending load), the maximum fracture displacement of the TBW model was small (0.27823 mm). However, when the knee joint was fully extended (0° axial load), probably owing to the constraint of the tension band, the fracture displacement of the patellar articular surface was larger. The fracture interface gradually separated, and the maximum fracture displacement of the TBW model reached a very dangerous value (1.9426 mm). Fracture space > 3 mm indicates the failure of internal fixation (Patel et al., 2000; Song et al., 2014). TBW also exhibited a higher stress concentration (approximately 1,310.8 MPa). Therefore, the K-wire tension band system alone may not provide sufficient compression in the early stage, especially when performing leg extensions. TBWC effectively reduces the structural stress and displacement and enhances the stability of the system; however, it exhibits similar biomechanical characteristics. Owing to the superficial position of the patella, complications associated with this system, such as implant irritation, postoperative pain, and delayed wound healing, cannot be ignored.

Therefore, metal-titanium cannulated screws are introduced when fracture conditions permit their use. According to previous reports, metal cannulated screws combined with TBW exhibit very strong rigid fixation strength, providing better stability and reducing the risk of fractures and dislocations (Liu et al., 2022). Lag screws can also produce sustained compression at patellar fracture sites. In the present study, TBWFS and TBWHS had good fixation results and produced continuous compressive stability at the fracture interface. Among them, TBWFS is the most stable, with the smallest fracture displacement during knee flexion and full extension (500 N; 0.074938, and 0.038759 mm, respectively), possibly due to the larger frictional contact area of the full-length thread. This provides a more comprehensive and lasting stability. The tension and compression effects of TBWHS result in a relatively large stress on the screws and patella. In addition, to avoid the high complication rate associated with TBW, some scholars avoid using TBW or replace it with suture treatment (Jia et al., 2022). However, re-surgery is often required to remove the internal fixation, and complications such as stress shielding also exist.

To promote the development of minimally invasive surgical treatments for the patella, we selected TBSAS for TPFs. Notably, several clinical reports have described this fixation regimen. Qi et al. (2011) reported that 12 months after patellar fracture fixation using two absorbable cannulated screws combined with a No. 5 Ethibond braid polyester suture tension band had an average Lysholm score of 95.7, and good clinical results were observed without any postoperative complications. Usami et al. (2021) reported that after patellar fracture fixation using two F-unsintered hydroxyapatite/poly-L-lactide screws and three FiberLoop sutures, there were no complications and a general return to the pre-injury level of work and activities of daily living. Biomechanical studies have also suggested that bioabsorbable implants demonstrate an efficacy comparable to that of metal prostheses in patellar fracture fixation (Adjal et al., 2022; Edoardo et al., 2022), consistent with the FEA results of the present study. At 500N, the maximum fracture displacement of TBWC (0.15633 mm) was similar to that of TBSAS (0.15272 mm) during knee flexion (45° bending load). No statistically significant difference was observed in the mean relative displacement of the fault ends. This further proves the feasibility of our finite element model. However, at full extension of the knee joint (0° axial load), TBSAS showed better data results than TBWC but close results to those of TBWFS and TBWHS. This may be due to the better axial holding capacity of the full-thread friction contact of the rigid screws. Furthermore, because of the material particularity of absorbable cannulated screws and high-strength sutures, the Young’s modulus of PLGA is closer to that of bone, and the suture material has better elasticity. Internal fixation and patellar stress results were lower in the TBSAS group. However, previous biomechanical studies have shown that the maximum load delivered by a knee extension device is approximately 316 N (Patel et al., 2000). Therefore, in the biomechanical experiments in the present study (0–500 N), although the stability of TBSAS was lower than that of TBWHS and TBWFS, it may be superior to that of TBW and comparable to or better than that of TBWC. Even under a stress load of 500 N, TBSAS exhibits good structural stability and stress loading, which meets the mechanical requirements of knee flexion and extension device movement, allowing early functional exercise.

Satisfactory results have been obtained in clinical trials. TBSAS is simple and flexible, with a shorter mean operative time than that of TBWC. In the clinical follow-up data, owing to each patient’s strict wound management and exercise guidance, no significant postoperative infection, internal fixation failure, revision, or other serious complications occurred in either patient group. However, in the early postoperative period, patients treated with TBSAS showed significantly smaller postoperative pain responses, shorter recovery-remission cycles, and lower VAS scores, which further affected the possibility of early functional exercises among patients. Notably, many patients in the TBSAS group achieved >100° knee flexion at 1 month postoperatively, which was more difficult to achieve in the TBWC group. This is consistent with the results of previous studies (Bai et al., 2021; Du et al., 2022). The Bostman score and final knee motion were slightly lower in the TBWC group than in the TBSAS group. Therefore, TBSAS demonstrated sufficient fixation strength and stability without significant abnormal complications, meeting the need for TPF fixation and early functional exercise. A single operation costs relatively high; however, it reduces metal-related complications and eliminates the pain associated with reoperation. In the present study, more patients were willing to undergo TBSAS treatment.

As a new type of internal fixation material, absorbable screws have unique advantages: 1) histocompatibility, degradability, non-toxicity, and low foreign body reaction; 2) Young’s modulus is closer to that of the bone and gradually degrades over a long period, resulting in adaptive stress transfer, promotion of bone growth, and prevention of stress shielding; 3) the fracture end can produce fretting (<0.5 mm) (Wang et al., 2023), which is conducive for fracture healing and reconstruction; and 4) the material begins to expand radially and shrink longitudinally 2 h after implantation, making the fixation firmer, and the initial fixation strength maintenance time can reach 3 months (Zhang et al., 2014). Combining absorbable screws with high-strength sutures using the tension band and “NICE” junction technologies can further strengthen the compression fixation of patellar fractures. The results of the biomechanical and clinical trials in the present study demonstrate their effectiveness and feasibility.

Notably, some researchers have developed simple suturing programs. Jirangkul and Kosiyatrakul (2021) demonstrated the stability and efficacy of Fiber Wire in treating TPFs through prospective clinical trials. Tang et al. (2018) used double 0-0 polydioxone sutures for braided five-pointed star lattice fixation. However, most of these studies were clinical cases, the follow-up duration was short, and plaster fixation was required for 2–3 weeks to ensure stability. Therefore, TBSAS treatment is more commonly recommendable for TPFs. Under the minimally invasive condition of reducing metal internal fixation-induced complications and secondary surgery-induced trauma, greater stability of the rigid fixation can be guaranteed to meet the need for early functional exercise of the knee joint.

Notably, TBSAS may be more appropriate for TPFs (AO/OTA 34 C1) and longitudinal patellar fractures (AO/OTA 34 B1.1/B2.1), whereas TBW and other suitable internal fixation regimens should be considered for complex comminuted patellar fractures.

This study had some limitations. First, biomechanical FEA was used to compare the mechanical trends of various internal fixation schemes. The construction of the models and the applied forces were simplified. Furthermore, the sample size of the clinical study was small, follow-up duration was insufficiently long, and prospective studies were lacking. In the future, we will completely reconstruct the kinematic structural model of the knee joint and incorporate the patellar force into the gait cycle for biomechanical studies. With better clinical patient follow-up, we will conduct in-depth biomechanical studies of screw absorption and fracture healing. We also intend to conduct a multi-center prospective clinical study was conducted to compare the clinical efficacy of several internal fixation schemes for treating TPFs.

## 5 Conclusion

Our biomechanical and clinical experiments showed that TBSAS is safe and effective for treating TPFs. It is insufficient compared to TBWHS and TBWFS; however, the fixation stability is comparable to that of TBWC, and it is also sufficient to meet the needs of fixation and early functional exercise for TPFs. Furthermore, it can effectively reduce metal internal fixation-induced complications and secondary surgery-induced trauma. Therefore, TBSAS is worthy of clinical application and promotion.

## Data Availability

The raw data supporting the conclusion of this article will be made available by the authors, without undue reservation.
