# Supplementary material for: Tension band high-strength suture combined with absorbable cannulated screws for treating transverse patellar fractures: finite element analysis and clinical study
Source: Front Bioeng Biotechnol. 2024 Mar 7;12:1340482. doi: 10.3389/fbioe.2024.1340482 (PMC10955129; doi:10.3389/fbioe.2024.1340482)
Supplement: Supplementary file 1 [file DataSheet1.DOCX]

The convergence analysis

The total number of elements was increased by globally reducing the edge length of the elements. Field variables such as von Mises stress and displacement are used as detection indexes. For example, Apply 45° 500N bending load to the top of the patella in the TBSAS model, the different field variables such as maximum of von Mises stress and displacement between 884983 and 2212660 nodes is less than 5% (88.29 ~ 88.31Mpa; 0.152~0.151mm), there was no maximum stress point, proving that the model reaches convergence.
